# Whole-body inhalation of nano-sized carbon black: a surrogate model of military burn pit exposure

**DOI:** 10.1186/s13104-022-06165-2

**Published:** 2022-08-11

**Authors:** Janeen H. Trembley, Simon W. So, Joshua P. Nixon, Elizabeth C. Bowdridge, Krista L. Garner, Julie Griffith, Kevin J. Engles, Thomas P. Batchelor, William T. Goldsmith, Julie M. Tomáška, Salik Hussain, Timothy R. Nurkiewicz, Tammy A. Butterick

**Affiliations:** 1 Minneapolis Veterans Affairs Health Care System, Minneapolis, MN USA; 2 Department of Laboratory Medicine and Pathology, University of Minnesota, Minneapolis, MN USA; 3 Masonic Cancer Center, University of Minnesota, Minneapolis, MN USA; 4 Department of Surgery, University of Minnesota, Minneapolis, MN USA; 5 Department of Physiology and Pharmacology, West Virginia University School of Medicine, Morgantown, WV USA; 6 Center for Inhalation Toxicology (iTOX), West Virginia University School of Medicine, Morgantown, WV USA; 7 Burn Pits 360 Veterans Organization, Robstown, TX USA; 8 Department of Food Science and Nutrition, University of Minnesota, St Paul, MN USA; 9 Department of Neuroscience, University of Minnesota, Minneapolis, MN USA; 10 Center for Veterans Research and Education, Minneapolis, MN USA

**Keywords:** Burn Pit Exposure, Chronic Multisymptom Illness, Inflammation, Cytokines, Environmental Exposure, Inhalation toxicology, Nanoparticle, Carbon Black

## Abstract

**Objective:**

Chronic multisymptom illness (CMI) is an idiopathic disease affecting thousands of U.S. Veterans exposed to open-air burn pits emitting aerosolized particulate matter (PM) while serving in Central and Southwest Asia and Africa. Exposure to burn pit PM can result in profound biologic consequences including chronic fatigue, impaired cognition, and respiratory diseases. Dysregulated or unresolved inflammation is a possible underlying mechanism for CMI onset. We describe a rat model of whole-body inhalation exposure using carbon black nanoparticles (CB) as a surrogate for military burn pit-related exposure. Using this model, we measured biomarkers of inflammation in multiple tissues.

**Results:**

Male Sprague Dawley rats were exposed to CB aerosols by whole body inhalation (6 ± 0.83 mg/m^3^). Proinflammatory biomarkers were measured in multiple tissues including arteries, brain, lung, and plasma. Biomarkers of cardiovascular injury were also assayed in plasma. CB inhalation exposure increased CMI-related proinflammatory biomarkers such as IFN-γ and TNFα in multiple tissue samples. CB exposure also induced cardiovascular injury markers (adiponectin, MCP1, sE-Selectin, sICam-1 and TIMP1) in plasma. These findings support the validity of our animal exposure model for studies of burn pit-induced CMI. Future studies will model more complex toxicant mixtures as documented at multiple burn pit sites.

## Introduction

Chronic multisymptom illness (CMI) is an idiopathic disease that thousands of U.S. military Veterans who served in Operations Enduring Freedom (OEF), Iraqi Freedom (OIF) and New Dawn (OND) suffer from daily. CMI is characterized by increased inflammation, chronic fatigue, musculoskeletal pain, impaired cognition, gastrointestinal disorders, respiratory problems, and skin rashes [1–6]. Burn pit exposures are a suspected cause for CMI. OEF/OIF/OND Veterans were exposed to toxic open-air burn pits. Waste burned included computer parts, animal carcasses, medical waste, lithium-ion batteries, plastics, Styrofoam, insecticide canisters, DEET-soaked items, human excrement, and vehicles. Collectively, the sites at large bases burned an estimated 85,000 pounds of waste per day, and burn pits were often located near military housing, work areas, and dining facilities [7, 8].

Burn pit emissions have been characterized to include fine particulate matter (PM_2.5_) with an average concentration above U.S. air pollution standards known to be associated with cardiovascular morbidity and mortality [9, 10]. Burn pit emissions contain a mixture of carcinogens, neurotoxins, and endocrine disruptors, toxins that are linked to chronic illness [11–13]. Inhalation toxicology research has shown that exposures to complex mixtures of gases and particles result in profound biologic consequences. Combined gases and solid particles create an aerosolized particle, forming a toxic delivery system. Pulmonary deposition of such toxicants initiates an inflammatory response characterized by oxidative stress, DNA damage, macrophage activation, autonomic stimulation, cell recruitment and chemokine production [14–17]. The result is production of “pulmonary shrapnel”: cellular and biochemical products that spill out of the lung and into the systemic circulation, leading to a cascade of short- and long-term biologic effects [18, 19].

We developed an animal model of burn pit-induced CMI using CB inhalation in rodents. Our rationale was to establish a simple exposure model, to which more complex toxicants may be added. Previous work in the Nurkiewicz lab has focused on metal oxide nanomaterial whole body inhalation studies in rodents, demonstrating negative impacts on systemic microvascular and cardiac function [18, 20–22]. Building upon the technological capabilities of the West Virginia University inhalation toxicology center, we used CB nanoparticles as an initial particle surrogate as carbon is a significant component of burn pit emissions. By developing this surrogate, a reliable and repeatable starting point for investigations into the pathology behind burn pit exposure-induced health problems will be established. We present data demonstrating that whole body CB inhalation induces multi-organ system inflammatory markers in rats, supporting this approach for modeling burn pit-induced CMI in US Veterans.

## Main text

### Methods

#### Carbon black nanoparticle preparation and characterization

Carbon black (CB) powder (Printex 90^®^, a gift from Evonik, Frankfurt, Germany) is composed of 99.9% carbon. CB aerosols were generated using a high-pressure acoustical generator (HPAG, IEStechno, Morgantown, WV). The output of the generator was fed into a Venturi pump (JS-60 M, Vaccon, Medway, MA) to further de-agglomerate the particles. The nano-CB aerosol/air mix was sampled in real-time with a light scattering device (PDR-1500, Thermo Environmental Instruments Inc., Franklin, MA) to estimate the aerosol mass concentration within the exposure chamber. Stable mass concentrations were maintained in real-time via software feedback loops. 37 mm PTFE filters were used for gravimetric measures concurrent with the PDR-1500 measures to obtain a calibration factor; gravimetric measures were also performed during exposures to calculate true mass concentrations. The particle count size distribution was measured using a high resolution electrical low-pressure impactor (ELPI, Dekati, Tampere, Finland). Particle mass size distribution of the CB aerosol was measured from the exposure chamber with a cascade impactor (Nano-MOUDI, 115R, MSP Corp, Shoreview, MN).

#### Electron microscopy

Aerosol characterization was verified throughout a given exposure by collecting CB particle samples on filters, and making hourly gravimetric measurements with a microbalance. This approach was also used to collect samples for transmission electron microscopy (TEM) and scanning electron microscopy (SEM).

#### Experimental animals

Male Sprague Dawley rats (8 weeks, 250–275 g) were obtained from Hilltop Laboratories (Scottdale, PA) and housed in an AAALAC approved facility at WVU. All procedures were approved by the WVU Institutional Animal Care and Use Committee (protocol 1602000621) and conformed to the most current National Institutes of Health (NIH) Guidelines for the Care and Use of Laboratory Animals. Housing conditions included 12:12 h light:dark cycle, 20–26 °C, 30–70% relative humidity, acclimatization for 48–72 h prior to any procedure and had ad libitum access to food and water.

#### Whole body inhalation exposure

Rats (sham-control group: age 53 ± 1 days; mass 288 ± 3 g. CB group age 53 ± 1 days; mass 303 ± 4 g.) were exposed to a target concentration of CB for 6 h: 6 mg/m^3^ (n = 10/group). Rats were randomly assigned to control or experimental groups and were individually housed in cages within a stainless steel chamber during exposure. The exposure chamber was 22″ × 20″ × 20″ (wdh) with an approximate volume of 144 L. The airflow through the chamber was approximately 28 LPM during exposures. Bedding material soaked with water is used in the exposure chamber to maintain comfortable humidity (30–70%) and temperature (20–26 °C) during the exposure. Sham-control animals were exposed to HEPA filtered air only.

#### Tissue collection and processing

Tissue harvesting were performed 24 h after exposure. Euthanasia was performed via exsanguination under deep anesthesia (5% induction, 2% maintenance with isoflurane gas) followed by organ removal. Plasma aliquots and whole tissues (arteries, brain, lung) were snap frozen in liquid nitrogen and stored at −80 °C. Solid tissues were pulverized on dry ice to granular powder. Tissue was homogenized with RIPA buffer (Thermo Fisher Scientific, Waltham, MA) or MILLIPLEX MAP buffer (MilliporeSigma, Burlington, MA). Total protein was determined by Direct Detect Spectrometer (MilliporeSigma).

#### Inflammation panel

The Meso Scale Discovery (MSD, Rockville, MD, USA) V-PLEX Rat Pro-inflammatory Panel 2 Kit (K15059G) was used to quantify tissue (50 μg) and plasma (25 µl) concentrations of IFN-γ, IL-1β, IL-4, IL-5 IL-6, IL-10, IL-13, KC/GRO, and TNF-α. All samples were run in duplicate. Plates were processed according to the manufacturer’s instructions using the MSD MESO Sector 600. Data were analyzed using MSD Discovery Workbench 4.0 software [5].

#### Vascular injury panels

Vascular injury was measured in plasma using the Rat Vascular Injury Panels 1 and 2 (RV1MAG-26 K & RV2MAG-26 K, MilliporeSigma). Samples were processed according to manufacturer recommendations, and data captured using Luminex Magpix (MilliporeSigma).

#### Data analysis

Biomarker data calculations were performed using GraphPad Prism 9. Differences between sham-control and CB exposed groups were identified by using unpaired *t*-test or Mann–Whitney tests according to data distribution (n ≥ 8 for all biomarkers per experimental group). Significance was established as p ≤ 0.05. Descriptive statistics are provided for CB aerosol characterization.

## Results

### Whole body CB inhalation exposure induces biomarkers of inflammation and vascular injury

**Fig. 1:**
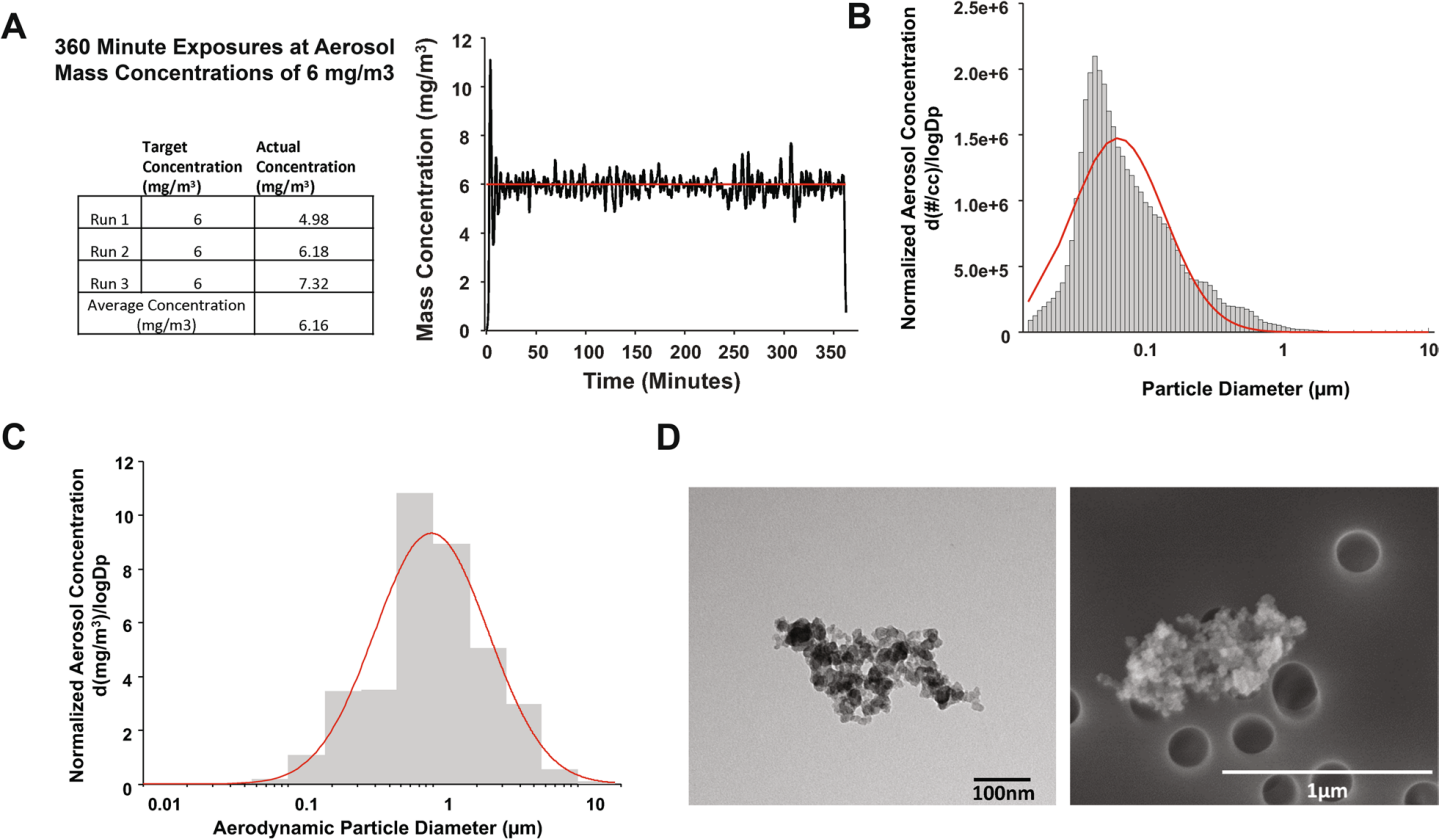
CB nanoparticle characterization and deposition in lung. **A** CB particle characterization table and real-time mass concentration measurements (mg/m^3^) of the CB aerosols during an inhalation exposure for 6 h for a target concentration of 6 mg/m^3^. Data represent an average of three exposures. **B** Particle size distribution of the carbon black aerosol measured from the exposure chamber using a high resolution electrical low-pressure impactor (ELPI +). A log-normal fit of the distributions resulted in a count median diameter (CMD) of 67 nm with a geometric standard deviation (GSD) of 2.13 nm. Inset: representative image of CB particles. Scale bar 500 nm. **C** Particle size distribution of the CB aerosol measured from the exposure chamber with a cascade impactor (Nano-MOUDI). A log-normal fit of the distribution resulted in a mass median aerodynamic diameter of 975 nm with a GSD of 2.47 nm. **D** TEM and SEM images of CB agglomerate collected on a TEM grid from the exposure 6 mg/m^3^ chamber

**Fig. 2:**
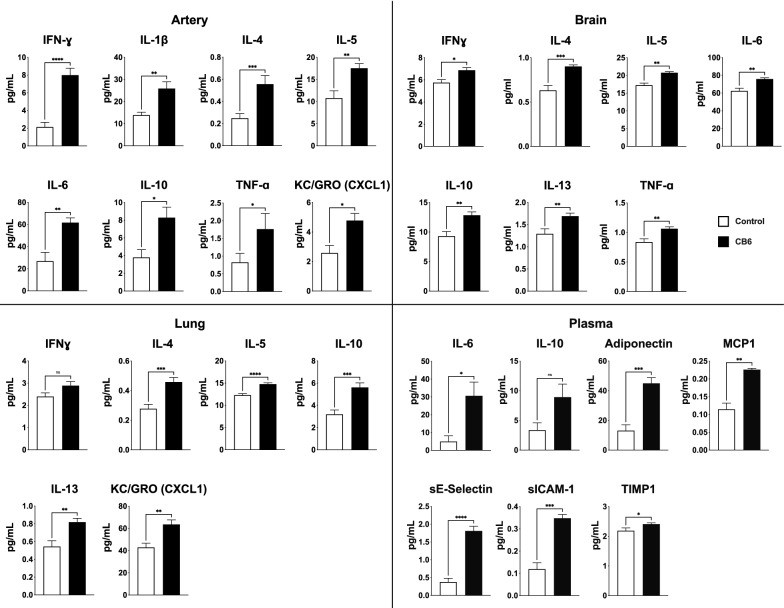
Pro-inflammatory and vascular injury biomarker analysis in rat tissues. Changes in tissue biomarkers of inflammation between control (white bars) and CB6 (grey bars) exposed rats are shown for all 4 tissues; vascular injury biomarkers are shown for plasma. **A** Artery. **B** Brain. **C** Lung. **D** Plasma. Data are represented as means ± SEM. Significance is denoted as: * p < 0.05, ** p < 0.005, *** p < 0.0005, ****p < 0.0001; ns, not significant at threshold of p < 0.05

Aerosolized CB particles are a particulate form of elemental carbon manufactured by the gas-phase pyrolysis and partial combustion of hydrocarbons. Use of whole-body inhalation exposure to CB represents a surrogate for particle exposure. Gravimetric data from the three exposures indicated an average aerosol mass concentration of 6.16 ± 0.83 mg/m^3^ (Target = 6 mg/m^3^). Real-time mass concentration measurements of the CB aerosols during a 6 h inhalation exposure are shown in Fig. 1A. CB aerosol particle count size distribution was measured from the exposure chamber using a high resolution electrical low-pressure impactor (ELPI +) and indicated a count median diameter of 67 nm with a geometric standard deviation (GSD) of 2.13 (Fig. 1B). Representative CB particle agglomerate TEM and SEM images are also shown (Fig. 1C). Particle size distribution of the CB aerosol in the exposure chamber was also measured with a cascade impactor (Nano-MOUDI). Based on a log-normal fit of the data, we calculated a mass median aerodynamic diameter of 975 nm with a GSD of 2.47(Fig. 2C). A representative image of CB deposited and captured on filter samples is shown in Fig. 1D.

Nine pro-inflammatory biomarkers were measured in artery, brain, lung, and plasma (Table 1). Eight markers of inflammation were significantly elevated in arteries of CB-exposed rats compared to sham-control rats: IFN-γ, IL-1β, IL-4, IL-5, IL-6, IL-10, KC/GRO (CXCL1), and TNFα (Fig. 2A). Seven inflammatory biomarkers were significantly elevated in whole brain tissue: IFN-γ, IL-4, IL-5, IL-6, IL-10, IL-13, and TNFα (Fig. 2B). In lung, the primary organ of impact following inhalation exposure, induction of four inflammatory biomarkers was observed: IL-4, IL-5, IL-10, and KC/GRO (Fig. 2C); IFN-γ trended toward elevation (p = 0.059). In plasma, IL-6 was significantly elevated (Fig. 2D), and IL-10 had a trend to be slightly elevated (p = 0.052). Using the vascular injury panels for analysis of plasma (Table 1), we observed significant increases in adiponectin, MCP-1, sE-Selectin, sICam-1 and TIMP1 (Fig. 2D).

**Table 1:** Pro-inflammatory and vascular injury biomarkers

Analyte	Function
IL-1β	Cytokine: potent mediator of the inflammatory response; innate Th2 inflammatory response
IL-4	Cytokine: innate Th2 inflammatory response
IL-5	Cytokine: essential for eosinophil differentiation and survival; innate Th2 inflammatory response
IL-6	Cytokine: pro-inflammatory roles; chronic inflammation
IL-10	Cytokine: induced by inflammation, role in limiting immune response
IL-13	Cytokine: innate Th2 inflammatory response
IFN-γ	Cytokine: important immunoregulatory functions; activator of macrophages
KC/GRO (CXCL1)	Chemokine: role in inflammation and as a chemoattractant for neutrophils
TNFα	Cytokine: innate Th2 inflammatory response
Adiponectin	Regulates glucose levels & fatty acid breakdown; positive association with cardiovascular mortality
MCP1 (CCL2)	Chemotactic for monocytes and basophils; associated with cardiovascular diseases and cognitive decline
sE-Selectin	Role in immunoadhesion; systemic endothelial dysfunction
sICAM-1	Role in immunoadhesion; systemic endothelial dysfunction
TIMP1	Regulator of extracellular matrix synthesis and degradation; biomarker of fibrosis

## Discussion

Data presented here support that our exposure model is a valid animal surrogate model for future studies on the effects of and treatment for military burn-pit related CMI. Deployment-related CMI compromises the health of thousands of our Veterans. Over 2.7 million U.S. military personnel were deployed in support of OEF, OIF and OND [23]. In a study to determine the frequency of CMI in OEF/OIF Veterans, it was found that at 1 year post-deployment over 60% met the criteria for mild or severe CMI [24]. Given the potential for short and long-term impacts on the health of US Veterans, modeling burn pit exposure to better understand the mechanisms of deployment-related illnesses is timely and critical. This initial study was set up as an acute exposure model with the goal of replicating deposition of burn pit particles. Our rationale for using a target concentration of 6 mg/m^3^ was to achieve an accumulated lung burden in this study to reflect longer-term exposures experienced by military personnel [25, 26]. While the actual lung burdens of deployed personnel will never be known, our conditions of exposure are consistent with what was reported in the various military burn pit sites.

Military environmental and occupational exposures are linked to elevated risk of cancer, pulmonary, cardiovascular, and metabolic diseases, and cognitive decline [8, 27–29]. Moreover, a wide array of health effects have been observed in humans and animals following exposure to specific air pollutants such as those found in military burn pits [13, 30–33]. Adverse health effects include eye and throat irritation, inflammation, pulmonary disease, and reduced or impaired cognitive function. Multiple organs and systems are known to be targeted by toxic airborne exposures, including blood, lungs, cardiovascular, and central nervous systems [34–37]. One of the most intensely tested concepts concerning health effects of exposure to airborne toxins is that the onset of inflammatory responses mediates the causal path from pulmonary exposure to development of pulmonary, cardiovascular, metabolic, malignant, and neurodegenerative diseases [29, 38, 39]. Here we present data from lung, plasma, artery, and brain demonstrating the inflammatory impact of inhaled PM relevant to military burn pit exposure.

For future studies, we propose to use more relevant mixtures of particles and gases in inhalation exposure systems. The WVU iTOX Center is fully equipped and currently performing multiple studies with mixed exposure models. Recent published work from the Hussain laboratory at WVU utilizing the mixed exposure model comprising of ozone and CB demonstrate a significantly greater decline of lung function capacity after mixed exposure compared to individual exposures [17]. Mechanistically, an oxidant mediated epithelial alarmin pathway was validated as a signaling mechanism in this model. Further, another study demonstrated increased reactivity to CB particles after interaction with ozone leading to a delayed endothelial monolayer wound repair through macrophage derived CXCR3 ligands [22]. These studies provide examples of robust evidence that novel mechanistic pathways are activated by particle and gas mixtures and establish a compelling rationale that support our hypothesis that mixed exposures initiate and/or drive CMI pathology. In the future, we propose incorporating complex mixtures with toxicants relevant to burn pit exposure such as volatile organic compounds, polyaromatic compounds, and polychlorinated dibenzo-p-dioxins (PCDDs) and dibenzofurans (PCDFs) [12, 40]. Prolonged and unresolved inflammation is proposed as a major contributor to the morbidity of chronic diseases such as CMI [3]. By modeling military burn pit exposures, subsequent studies will be directed toward understanding the progression from acute inflammation to compromised systemic resolution and the resultant development of chronic inflammatory disease such as CMI.

This model has wider applicability for occupational- and pollution-related exposure studies of particle inhalation and human health impact. Exposures to toxins in burn pit smoke are similar to exposures experienced by first responders and military personnel, and first responder occupations are linked to altered immune and inflammatory markers and activity associated with development of chronic diseases [38]. Previous work in the Nurkiewicz laboratory has focused on rodent whole body nanomaterial inhalation studies associated with occupational and related hazards [31, 41, 42]. Here, we have built upon this body of work to develop a surrogate model of military burn pit exposure. We used CB, a significant component of military burn pit emissions and aerosolized particulate matter, as the starting point for investigations into the pathology behind burn pit exposure-induced health problems such as CMI. Our results provide evidence that whole-body inhalation exposure to CB induces a proinflammatory biomarker signature in multiple tissues (artery, brain, lung, and plasma) and biomarkers of cardiovascular injury in plasma.

## Limitations

- Use of single toxicant and not a complex mixture
- Study focused on short-term exposure
- Limited scope of biomarker screening
- Male rats only

## Data Availability

The datasets generated during and/or analyzed during the current study are available from the corresponding author on reasonable request.
